# Ultraviolet Laser Damage Dependence on Contamination Concentration in Fused Silica Optics during Reactive Ion Etching Process

**DOI:** 10.3390/ma11040577

**Published:** 2018-04-10

**Authors:** Laixi Sun, Ting Shao, Zhaohua Shi, Jin Huang, Xin Ye, Xiaodong Jiang, Weidong Wu, Liming Yang, Wanguo Zheng

**Affiliations:** 1 Research Centre of Laser Fusion, China Academy of Engineering Physics, Mianyang 621900, China; sunlaixi@yahoo.com (L.S.); shaotingcaep@163.com (T.S.); shizhaohuacaep@163.com (Z.S.); yanglimingzs@163.com (L.Y.); 2 Science and Technology on Plasma Physics Laboratory, Research Center of Laser Fusion, China Academy of Engineering Physics, Mianyang 621900, China; jiangxiaodongzr@163.com; 3 IFSA Collaborative Innovation Center, Shanghai Jiao Tong University, Shanghai 200240, China; wuweidonging@163.com (W.W.); zhengwanguosz@163.com (W.Z.)

**Keywords:** fused silica, contamination, reactive ion etching, laser damage, optical performance

## Abstract

The reactive ion etching (RIE) process of fused silica is often accompanied by surface contamination, which seriously degrades the ultraviolet laser damage performance of the optics. In this study, we find that the contamination behavior on the fused silica surface is very sensitive to the RIE process which can be significantly optimized by changing the plasma generating conditions such as discharge mode, etchant gas and electrode material. Additionally, an optimized RIE process is proposed to thoroughly remove polishing-introduced contamination and efficiently prevent the introduction of other contamination during the etching process. The research demonstrates the feasibility of improving the damage performance of fused silica optics by using the RIE technique.

## 1. Introduction

Since subsurface damage (SSD) of fused silica optics introduced by sorts of surface finishing processes has been determined to be responsible for a majority of ultraviolet laser induced damage (LID) [1,2,3], many stringent fabrication and modification methods are required to improve the laser-induced damage threshold (LIDT) of fused silica. Unfortunately, the damage precursors in the subsurface layer such as scratches and embedded impurities are too stubborn to be avoided, even though the surface fabrication level of fused silica optics nearly approach to its limit. Recently, more efforts have been focused on removing the SSD by developing advanced surface modification processes, such as HF-based etching [4,5], ion beam etching (IBE) [6,7,8], magnetorheological polishing (MRF) [9] and reactive ion etching (RIE) [10,11]. Comparing the different attempts at surface modification for fused silica, the last-mentioned RIE technique exhibits some distinct advantages. First, unlike HF-based wet etching, the dry etching can provide highly anisotropic etching profiles thus achieving significant removal of structural defects in the subsurface layer [12]. Second, the optical surface is rarely subjected to energetic ion-bombardment causing injection and damage, in contrast, for example, to the surface treated by IBE [13]. Third, the non-contact method does not involve direct contact contamination such as with carbonyl iron particles, which is inevitably used in the MRF process [14].

Historically, RIE has been known for decades as a micro- and nanostructures fabrication method of silicon-based material due to its capability for providing highly anisotropic etching profiles with good selectivity [15,16,17,18]. However, very few studies have been reported on how to increase the LIDT of fused silica by using the RIE technique. Juškevičius has used an argon plasma etching method for improving the laser-induced damage resistance of fused silica. However, the etching efficiency is very limited due to the etching process actually not involving a chemical reaction [19]. For RIE of fused silica, a key challenge is overcoming the surface contamination of fused silica during the etching process, including injected metal impurity and carbonaceous deposit [20,21].

In this work, we investigate the detailed evolution of contamination level in fused silica surfaces as a function of RIE depth. Particularly, the dependences of contamination concentration on LIDT are evaluated and explained comprehensively by changing the important processing parameter of RIE. Additionally, an attempt at eliminating the chance of contamination deposition on a fused silica surface during the RIE process is done by optimizing the plasma generating conditions including discharge mode, etchant gas and electrode material. Through further experiment, contamination on the optical surface of fused silica is prevented efficiently, which drives the improvement of LIDT.

## 2. Theoretical Details

**Figure 1:**
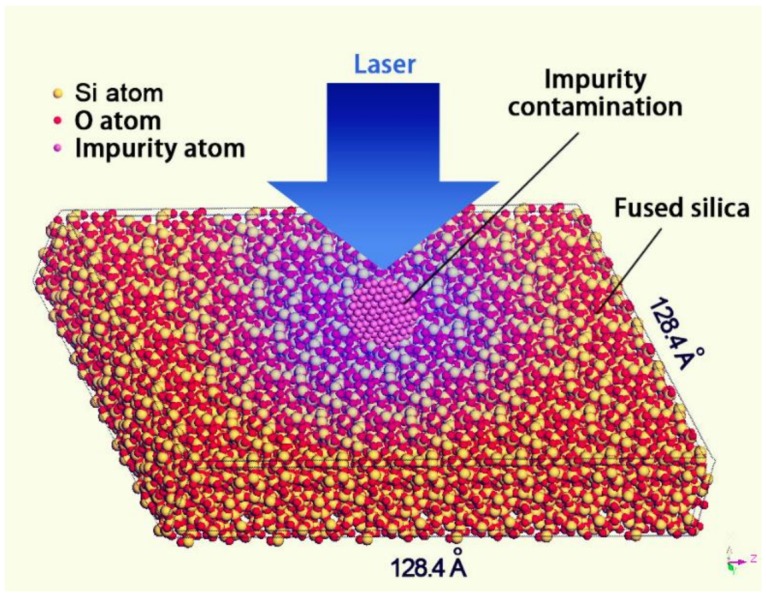
Schematic diagram of damage initiation induced by an impurity particle in fused silica optical surface.

**Figure 2:**
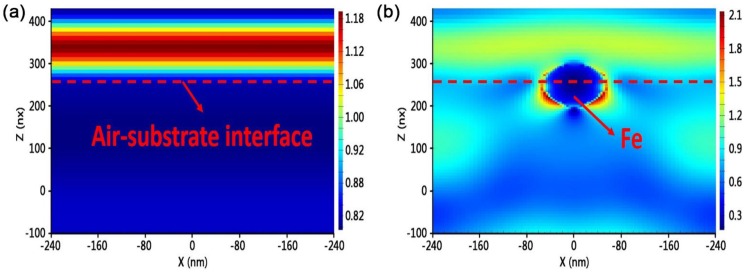
Simulation of intensity distribution of electric field (V/m) with and without impurity particle under the same boundary conditions for (**a**) contamination-free surface; (**b**) surface contaminated by a Fe impurity particle. The Light is vertically incident from top and the wavelength is 355 nm.

Surface contamination of fused silica is a critical factor influencing optical performance, which ranges from being burdensome, as obstacles resulting in downstream intensity modulation of laser light, to catastrophic, as precursors of laser induced damage. Lattice heating induced by multi-photon absorption has been shown to be responsible for the damage occurrence [22] and a model of small-absorber initiated damage has been demonstrated, which indicates that the damage can occur when absorbing defects, such as impurity particles, reach a critical temperature [23]. The diameter of impurity particles leading to LIDT decrease can even be at the nanometer scale [24]. Figure 1 shows a schematic diagram of damage initiation induced by an impurity particle in fused silica surface. The particle absorbs sub-band gap light and locally heats the defect-free silica surrounding it. The bulk begins to absorb laser energy through temperature-activated intrinsic absorption once the particle reaches a sufficient temperature. This finally leads to absorption run-away which drives the bulk material to extremely high temperatures and pressures resulting in explosive material ejection from the surface. The corresponding mechanism has been described in References [25,26]. In addition to absorption-induced damage, contamination by high refractive index or opaque impurities in the input surface of fused silica optics has been shown to lead to downstream light modulation with an intensity high enough to damage the optical surface [27,28]. The simulation result (shown in Figure 2) by the finite difference time domain method shows that the intensity of the electric field along the edge of the Fe impurity particle was enhanced by ~260% (see Figure 2). This model allows us to predict the threat to optics due to input surface contamination. Thus, more attention should be paid to investigate and remove the impurity contamination which can cause laser damage of fused silica.

## 3. Investigation Experiment of Contamination

In the investigation experiment, four fused silica samples were prepared from polished 50 mm round optics (Corning 7980). Cerium oxide slurries were used in the grinding and polishing process for all the samples. The RIE process was conducted in a self-developed inductively coupled plasma (ICP, 13.56 MHz) discharge system using a CF_4_-Ar mixture with an input power and chamber pressure of 200 W and 20 mTorr, respectively. All the samples (Marked as 1, 2, 3 and 4) were first cleaned using deionized water and then air dried. Then samples 2, 3 and 4 were treated by the RIE process with etching depths of 2 μm, 6 μm and 10 μm, respectively. The gas flow ratio of CF_4_ and Ar was 1:1 and the etching rate is about 2 μm/h. The detail of the samples preparation is given in Table 1.

Contamination levels as a function of embedding depth in the sample surfaces were studied by a Time of Flight Secondary Ion Mass Spectrometry (ToF-SIMS). The data were normalized with the concentration of silicon element (counts 10,000) as a standard. All sample surfaces were sputtered for 20 s to remove the contamination introduced by the surroundings. Surface morphology of the samples was observed by an optical microscope and surface absorption was characterized by a photo-thermo weak-absorption test apparatus based on thermal-lens elements with the wavelength of continuous pump laser being 355 nm. In order to investigate the dependence of LID on contamination levels, a damage test was performed with an ultraviolet laser (355 nm) with a beam diameter of 3 mm and the near-Gaussian pulse width of 9.5 ns. The test was performed using R-on-1 irradiation (damage threshold responses to incrementally ramping the laser fluence) focusing on the rear surface of the samples. The description of the laser system is given in detail elsewhere [29].

**Figure 3:**
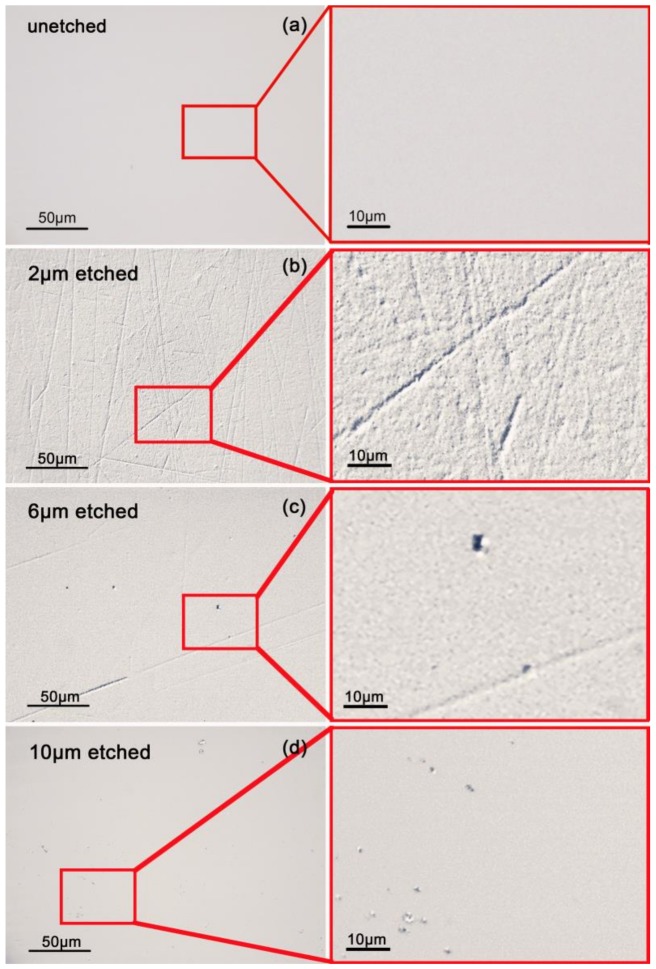
Surface morphologies with different RIE depths for (**a**) unetched sample; (**b**) 2-μm-etched sample; (**c**) 6-μm-etched sample; (**d**) 10-μm-etched sample.

Figure 3 exhibits the surface morphologies of the samples with different etching depths. There are not any defects (scratches or contaminations) on the unetched sample surface and the surface is very clean and smooth (see Figure 3a). Compared with the unetched sample, numerous microscopic scratches were exposed when the removal amount was 2 μm (see Figure 3b). The sample surface presented a rough microscopic texture and the width of some scratches even approached up to micron scale. In contrast, the sample with a 6 μm etching depth (see Figure 3c) presented a smoother and cleaner surface. Almost all the scratches were removed by the etching and rare scratches with larger width and depth remained on the sample surface. Particularly, the sample with 10 μm etching depth (see Figure 3d) presented a much smoother and cleaner surface. The results suggested that the structural defects in the subsurface layer could be removed effectively by RIE treatment so as to smooth the optical surface of fused silica.

**Figure 4:**
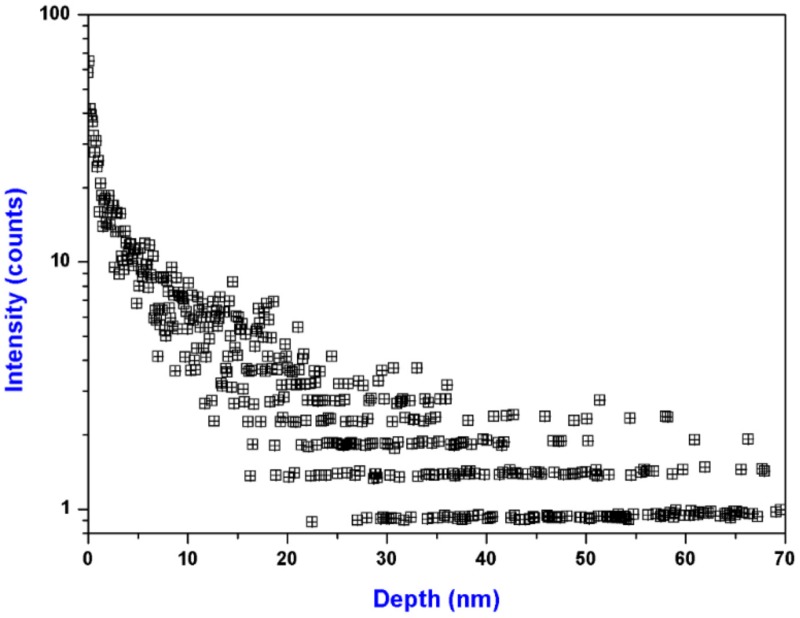
Depth profile of Ce in the sample surfaces. No Ce signal was detected on all the etched sample surfaces, thus not shown in the figure.

The polished surface of fused silica generally has a redeposition layer with the thickness of approximately 100 nm. Impurities associated with the polishing deposition, most notably ceria, are known damage precursors of low threshold. Figure 4 shows the depth profile of Ce on an unetched sample surface. The Ce impurity was enriched at the near-surface and finally dropped to zero as an exponential decay with the detecting depth increased. No Ce signal was observed on all the etched sample surfaces, thus it is not shown in the figure. The results suggest that the Ce contamination on the unetched sample surface is introduced by the polishing process. The polishing powder can only affect the shallow layer of the sample surface, so the Ce concentration in the bulk of fused silica is very low. And there is no any contamination source during the RIE process, so we did not find any Ce element on the RIE-treated sample surfaces.

**Figure 5:**
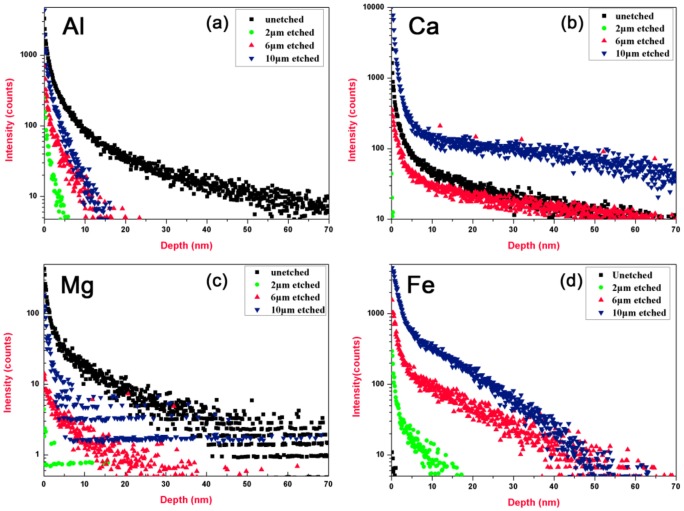
Depth profiles of impurity elements in sample surfaces with different etching depths for (**a**) Al; (**b**) Ca; (**c**) Mg; (**d**) Fe.

The ToF-SIMS testing results indicated Ce was not the only, or even the major impurity in the redepositon layer. Figure 5 shows the depth profiles of Al, Ca, Mg and Fe as a function of detecting depth on the sample surfaces. The peak concentrations of these impurities on the unetched sample (sample 1) surfaces were also located at the near-surface and then fell off sharply as the detecting depth increased. Unlike the evolution of Ce concentration in Figure 4, there are some interesting phenomenon observed from the results in Figure 5. First, all the impurities could not be completely eliminated by the etching treatment and the concentrations of these impurities were increased with the etching depth, suggesting that the etching treatment could introduce impurities. Second, the contamination level was varied in element type. For example, the concentrations of Al and Mg on the etched sample surfaces were all lower than that on the unetched sample surface (see Figure 5a,c). However, we noted that the 10 μm etched sample presented a higher concentration of Ca, comparing with the unetched sample (see Figure 5b). We also surprisingly found that the concentrations of Fe on all the etched sample surfaces were higher than that on the unetched sample surface, as shown in Figure 5d. The results further suggested that the etching treatment could introduce impurities, though the polishing-introduced contaminations could be effectively removed accompanying by the removal of SSD. We believed that the etching-introduced impurities might result from the physical bombardment of the inner wall of the chamber or the sample stage when energetic ions were accelerated by sheath bias of the plasma.

**Figure 6:**
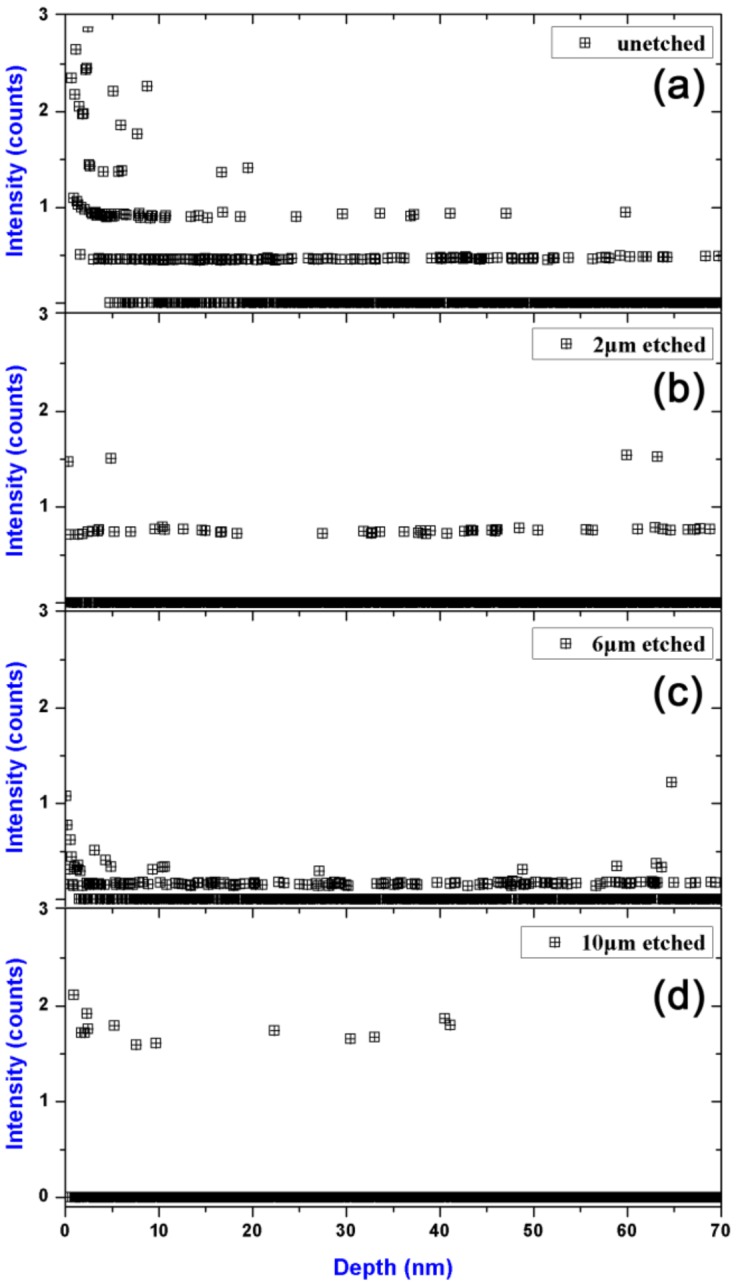
Depth profiles of C in the sample surfaces with different etching depths for (**a**) unetched sample; (**b**) 2-μm-etched sample; (**c**) 6-μm-etched sample; (**d**) 10-μm-etched sample.

**Figure 7:**
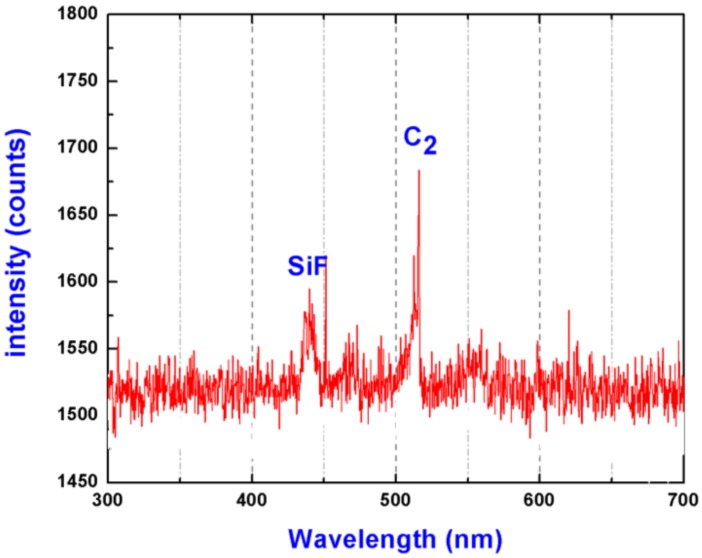
Atomic emission spectrum of CF_4_-Ar plasma near the sample surface.

Since fluorocarbon-based plasma etching process often accompany by fluorocarbon polymer deposition, carbon contamination is often found in the deposited films [30,31]. Previous studies have demonstrated carbon-contained deposit on fused silica surface could develop catastrophic damage growth with laser irradiation shot increased [20]. To determine if fluorocarbon polymer was deposited on the sample surfaces during the RIE process, the depth profiles of C with different etching depths were also investigated, as shown in Figure 6. Noted that there is no obvious carbon signal observed either on the unetched or etched sample surfaces. The results can be understood by the following two hypotheses: (1) fluorocarbon polymer deposit did not form on the sample surfaces during the RIE process; (2) fluorocarbon deposition occurred but the deposit was bombarded off by the energetic ions during the etching process. To figure out the nature, a typical atomic emission spectrum was obtained by diagnosing the CF_4_-Ar plasma near the sample surfaces, as shown in Figure 7. Compared with the essential intermediate specie SiF, which plays the role of forming SiF_4_, the C_2_ specie has a much higher intensity, which has been demonstrated as an important growth precursor of fluorocarbon polymer deposition [32]. We thus believed fluorocarbon polymer, in the present CF_4_-Ar plasma generating condition, could readily deposit on the sample surfaces but thoroughly be bombarded off by the energetic ions.

**Figure 8:**
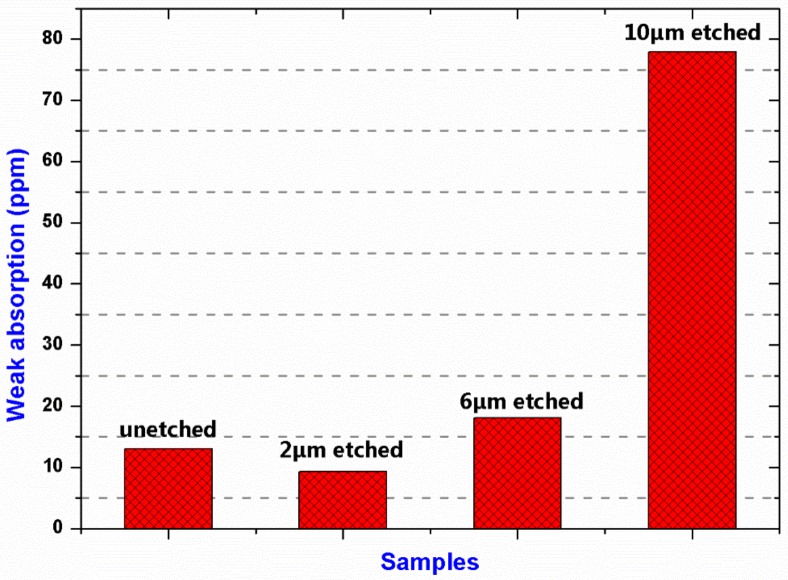
Surface weak absorption of the samples with different etching depths.

Prior to investigating the damage performance of the samples, the surface absorption was measured by a photo-thermo weak-absorption test apparatus. Figure 8 shows the surface weak-absorption of the samples with different etching depths. The surface absorption of the 2 μm etched sample (~9 ppm) was a little lower than that of the unetched sample (~13 ppm). However, obvious absorption enhancement has been observed on the deeper-etched sample surfaces, especially on the 10 μm etched sample surface. The weak-absorption results could be explained by the aforementioned absorption theory in Section 2. Impurities could be sputtered from the chamber inner wall or the sample stage onto the sample surfaces during the RIE process. At a relatively low etching depth (2 μm), except for a little increase in Fe concentration, the etched surfaces had no obvious increase in impurity concentrations of Al, Ca and Mg comparing with the unetched sample surface. In addition, Ce element was removed thoroughly following the etching. At a relative high etching depth (6 and 10 μm), the concentrations of Al, Ca, Mg and Fe were increased sharply. The impurity particles absorbed sub-band gap light and locally heated the fused silica. When the silica material around the impurity particles reached a sufficient temperature, the bulk began to absorb laser energy through temperature-activated intrinsic absorption. From the present results, however, it was difficult to determine which of these impurity elements was dominant for the absorption enhancement of the sample surfaces. But it could be inferred that their combination would have a neglect effect on the damage performance of the fused silica optics.

**Figure 9:**
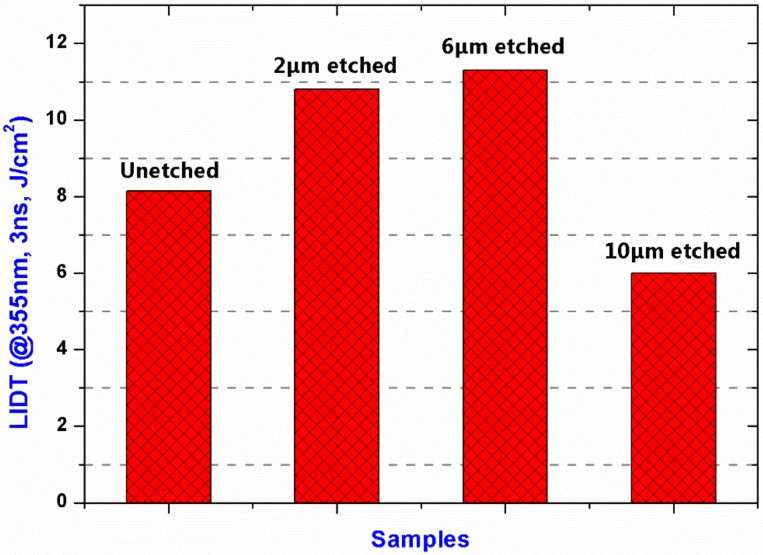
Laser-induced damage threshold (LIDT) of the samples with different etching depths.

The LIDT test of the samples with different etching depths was performed, as shown in Figure 9. Compared with the unetched sample, the LIDT of the 1 μm etched sample increased from 8.1 to 10.9 J/cm^2^. It was possibly due to the removal of the impurities such as Ce, Ca and Mg. When the etching depth increased to 6 μm, the LIDT had no obvious increase suggesting etching-introduced impurities began to play a more important role in damage than SSD. Particularly, there was a sharp decrease in LIDT (~6 J/cm^2^) at the etching depth of 10 μm. It was possibly because of the increase of the etching-introduced impurities, especially Fe, causing a strong laser absorption and then damage initiation. The results agreed well with the testing results by ToF-SMIS and weak-absorption, suggesting RIE-introduced impurities was a key factor for improving the LIDT of fused silica.

Summarized from the above investigating experiment results, we can find that the etching-introduced impurities in fused silica surfaces seriously influence the optical performance of the optics. To obtain an ideal optical surface with high LIDT, etching-introduced impurities have to be efficiently controlled or removed. Especially, some kinds of mental impurities may be dominant for the absorption enhancement of the sample surfaces. Fortunately, mental impurities in subsurface layer of fused silica can be efficiently removed by chemical leaching in strong acid solution such as HNO_3_ or HCl et al. [33]. We can also optimize the RIE process to reduce the concentration of etching-introduced impurities on fused silica surface, which will be represented below. Additionally, the existence of C_2_ species is still a hidden trouble for achieving high LIDT of fused silica, although the etched surface has no obvious fluorocarbon deposit under the present plasma generating condition. Last but not least, the removal rate of the SSD is seemingly insufficient since the structural scratches remain in the 6 μm etched sample surface. In short, the plasma generating condition has to be optimized and improved.

## 4. Optimization Experiment of Contamination

In the optimization experiment, we have attempted to eliminate the chance of contamination deposition on fused silica surface during RIE process, while enhancing the removal rate of SSD by improving the plasma generating condition.

### 4.1. Optimization Details

The optimization experiment involves in the following key steps:

(1) Instead of the previous discharge mode (ICP), the plasma source was changed to be capacitive coupled plasma (CCP) discharge mode [34,35]. It provided much lower plasma density and much higher ion energy, which could achieve more anisotropic SSD removal. Under this discharge condition, fluorocarbon deposition on fused silica surfaces could be avoided more efficiently. More importantly than that, the plasma transport became more perpendicular to the fused silica surface. In the case, the probability of ion bombardment onto the chamber inner wall would be reduced dramatically so as to prevent the impurity contamination on the optical surface.

(2) To further enhance the anisotropic removal rate of SSD and control the fluorocarbon deposition, H was added to the CF_4_ by using CHF_3_. Hydrogen atoms, which is an important initial neutral species deriving from collisions between electron and CHF_3_ molecule, react through F abstraction reaction forming relatively inactive HF acid as well as unsaturated C bonds. Simultaneously, O evolved in the etching of SiO_2_ competes with fluorocarbon polymerization reaction by combining with C to form volatile products such as CO, CO_2_ or even COF_2_, thereby allowing SiO_2_ etching to be continued. The corresponding chemical-reaction processes was described by the following equations (the gas-phase reaction of CHF_3_ plasma is described in detail elsewhere [36,37]):

CHF_3_ + e^−^ → CF^+^ + H + 2F + 2e^−^, (1)

CF^+^ + e^−^ → C + F, (2)

F + H → HF,(3)

C + O → CO,(4)

C + 2O → CO_2_, (5)

C + O + 2F → COF_2_, (6)

Additionally, the gas flow proportion of Ar in the gas mixture was doubled to enhance the energetic ion bombardment. The surfaces of the samples could probably be smoothed significantly due to the enhanced anisotropic etching. The corresponding mechanism of surface smoothing for fused silica during reactive ion or ion beam etching process has been described in Refs. [7,12].

(3) Once the discharge mode is charged to CCP, the physical bombardment on the sample stage was enhanced. Thus, the substrate was necessarily covered by a graphite cover-plate to avoid metal impurity being sputtered from the metal substrate onto the sample surface causing contamination and damage. Graphite, whose melting point is very high, has a stable chemical property. Thus, C was more difficult to be physically sputtered and form fluorocarbon deposit on fused silica surface. In addition, the strong physical bombardment could efficiently remove the possible fluorocarbon deposit on the optical surface of fused silica.

### 4.2. Results and Discussions

The etching experiments were all performed in the optimized plasma discharge system using CHF_3_-Ar mixture with the gas flow ratio of 1:2. During the etching, all the input parameters including the input power, chamber pressure and etching rate were almost the same as before. To ensure the comparability with the previous results, LIDT test was performed by using the same damage-testing platform with the same laser parameters and testing method.

**Figure 10:**
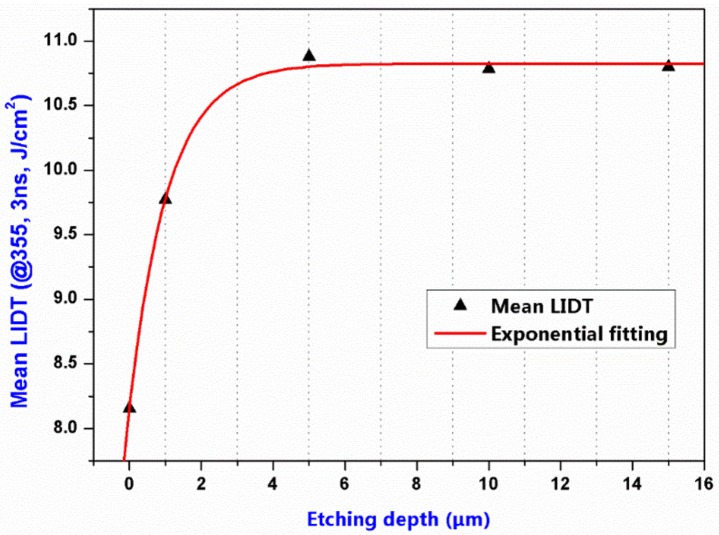
Mean LIDT as a function of etching depth by the optimized RIE process.

Figure 10 shows the fitting curve of mean LIDT as function of etching depth. Although there was no obvious improvement of LIDT at 6-µm etching depth comparing with the previous 6-µm-etched sample (Sample 3), the decrease of LIDT at 10-µm etching depth did not occur under the present etching condition. It suggested that the RIE-introduced impurity contamination was efficiently controlled in the optimization experiment. In addition, further increase in LIDT was limited when etching depth exceeds 6 µm suggesting that deeper etching may induce some other trace defects which has been demonstrated in Ref. [10]. Here, taking space limitation into consideration, we only analyzed the results of the unetched and 10-µm-etched sample marked as 1s and 4s respectively (see Table 2).

**Figure 11:**
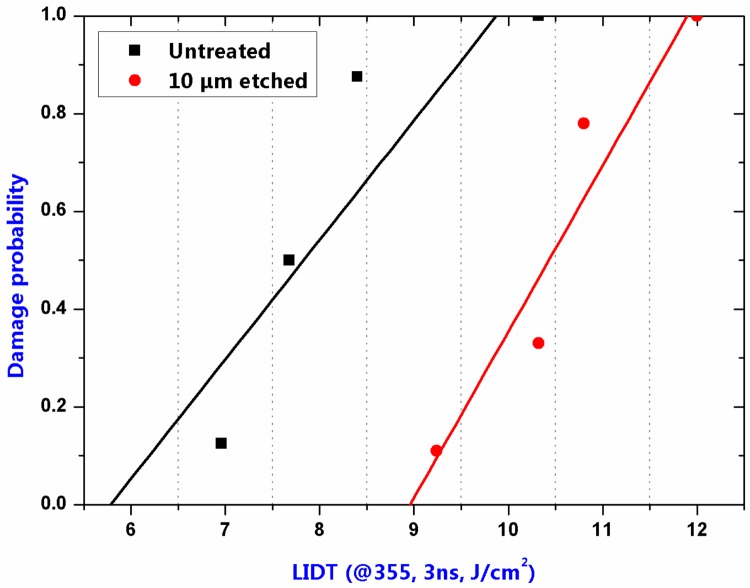
Damage probability of the unetched and 10-µm-etched samples.

The relationship between LIDT and damage probability of the two samples are shown in Figure 11 and Table 2. Compared with the unetched sample (Sample 1s), the damage thresholds of 0% probability and 100% probability of the 10-µm-etched sample (Sample 4s) were both increased dramatically suggesting the damage precursors in the subsurface layer were significantly removed. More conspicuously, the phenomenon that the mean LIDT sharply decreased at the 10-µm etching depth under the previous discharge condition did not occur on the sample with the same etching depth under the present discharge condition. We thus believe that the etching-introduced impurity contamination can be efficiently prevented after the optimization of the plasma generating condition.

**Figure 12:**
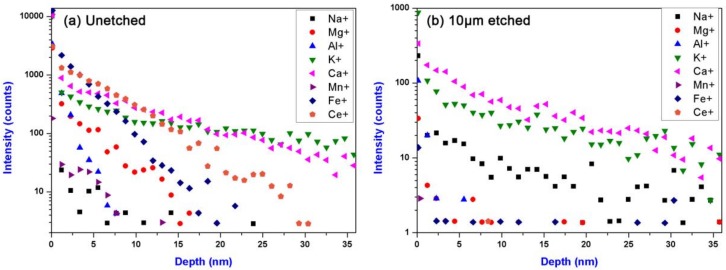
Depth profiles of the impurity elements in sample surfaces for (**a**) unetched sample; (**b**) 10 µm-etched sample.

The depth profiles of impurity elements in the sample surfaces with different etching depths were investigated, as shown in Figure 12. More comprehensive element types were detected to obtain the explicit understanding about the influence level of different kinds of impurity elements on damage performance of fused silica. It can be noted that there were also massive impurities in the redeposition layer of the unetched sample, including K, Ca, Ce, Al, Mg, Fe, and so forth. In contrast, except the alkali metal elements (K, Na and Ca), the concentrations of all other impurities of the 10-µm-etched sample were dramatically decreased with the detecting depth increases and their peak concentrations decreased by nearly 2 orders of magnitude (from ~10^5^ to ~10^2^). The results suggested that the optimized RIE process could efficiently prevent the etching-introduced impurity contamination, especially the typical photoactive metal impurities such as Ce, Al, Fe, and so forth. According to the damage results in Figure 9 and Figure 12, it can also be indicated that the alkali metal elements had weak influence on the LIDT of fused silica.

**Figure 13:**
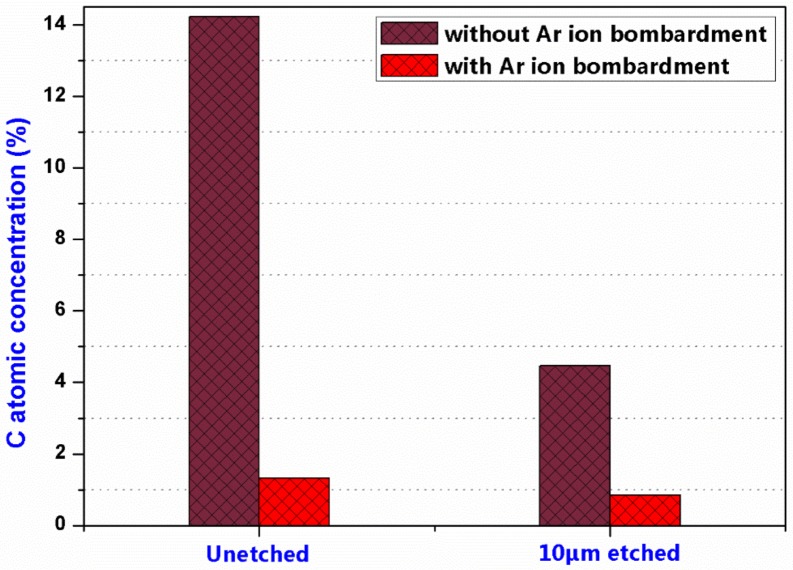
Atomic concentration of C in the sample surfaces.

X-ray photoelectron spectroscopy (XPS) measurement was conducted to investigate the C atomic concentrations on the sample surfaces according to the core spectra of XPS in order to distinctly validate the controlling capability of fluorocarbon contamination during the optimized RIE process. The results shown in Figure 13 included the C atomic concentrations on unetched and 10-μm-etched sample surfaces with and without Ar ion bombardment to obviate the environmental influence. Noted that there was an obvious decrease in C concentrations on the 10-μm-etched sample surface, no matter Ar ion bombardment was used or not. Comparing with the samples without Ar ion bombardment, there was tiny C residue (which can be neglected due to the minimal amount) on the sample surfaces which were bombarded by Ar ion. The results suggested that the surface contamination of C could be efficiently prevented during the optimized RIE process.

## 5. Conclusions

Reactive ion etching process of fused silica often accompanies by surface contamination, which seriously influences the ultraviolet laser damage performance of the optics. Through theoretical analysis and experimental investigation, we find that the RIE process is very sensitive to the contamination behavior and can be optimized by changing the plasma generating conditions such as discharge mode, electrode material and etchant gas. Additionally, an etching experiment using the optimized process is proposed to thoroughly remove the polishing-introduced contamination and efficiently prevent from introducing other contamination during the etching process. The results indicate that the optimized etching process can efficiently improve the LIDT of fused silica optics.

## Figures and Tables

**Table 1:** Samples preparation by reactive ion etching (RIE).

No	RIE Etching	Etching Depth (μm)
1	No	0
2	Yes	2
3	Yes	6
4	Yes	10

**Table 2:** Damage threshold of the unetched and 10-µm-etched samples.

No.	Etching Depth (μm)	0% Probability Damage Threshold (J/cm^2^)	100% Probability Damage Threshold (J/cm^2^)	Mean LIDT (J/cm^2^)
1s	0	5.8	9.9	8.2
4s	10	9.0	11.9	10.9
